# MYBL2 disrupts the Hippo-YAP pathway and confers castration resistance and metastatic potential in prostate cancer

**DOI:** 10.7150/thno.56604

**Published:** 2021-03-31

**Authors:** Qiji Li, Min Wang, Yanqing Hu, Ensi Zhao, Jun Li, Liangliang Ren, Meng Wang, Yuandong Xu, Qian Liang, Di Zhang, Yingrong Lai, Shaoyu Liu, Xinsheng Peng, Chengming Zhu, Liping Ye

**Affiliations:** 1 Department of Orthopaedic Surgery, The Seventh Affiliated Hospital, Sun Yat-sen University, Shenzhen, 518107, China.; 2 Department of Orthopaedic Surgery, The First Affiliated Hospital of Sun Yat-sen University, Guangzhou, 510080, China.; 3 Department of Urology, The Seventh Affiliated Hospital, Sun Yat-sen University, Shenzhen, 518107, China.; 4 Clinical Experimental Center, Jiangmen Central Hospital, Affiliated Jiangmen Hospital of Sun Yat-sen University, Jiangmen, 529030, China.; 5 Department of Experimental Research, State Key Laboratory of Oncology in South China, Collaborative Innovation Center for Cancer Medicine, Sun Yat-sen University Cancer Center, Guangzhou, 510060, China.; 6 Scientific Research Center, The Seventh Affiliated Hospital, Sun Yat-sen University, Shenzhen, 518107, China.; 7 Department of Pathology, The First Affiliated Hospital of Sun Yat-sen University, Guangzhou, 510080, China.

**Keywords:** Prostate cancer, Castration resistance, Hippo-YAP pathway, RhoA activation, MYBL2.

## Abstract

**Rationale:** Resistance to androgen-deprivation therapy (ADT) associated with metastatic progression remains a challenging clinical task in prostate cancer (PCa) treatment. Current targeted therapies for castration-resistant prostate cancer (CRPC) are not durable. The exact molecular mechanisms mediating resistance to castration therapy that lead to CRPC progression remain obscure.

**Methods:** The expression of MYB proto-oncogene like 2 (MYBL2) was evaluated in PCa samples. The effect of MYBL2 on the response to ADT was determined by *in vitro* and *in vivo* experiments. The survival of patients with PCa was analyzed using clinical specimens (n = 132) and data from The Cancer Genome Atlas (n = 450). The mechanistic model of MYBL2 in regulating gene expression was further detected by subcellular fractionation, western blotting, quantitative real-time PCR, chromatin immunoprecipitation, and luciferase reporter assays.

**Results:** MYBL2 expression was significantly upregulated in CRPC tissues and cell lines. Overexpression of MYBL2 could facilitate castration-resistant growth and metastatic capacity in androgen-dependent PCa cells by promoting YAP1 transcriptional activity via modulating the activity of the Rho GTPases RhoA and LATS1 kinase. Importantly, targeting MYBL2, or treatment with either the YAP/TAZ inhibitor Verteporfin or the RhoA inhibitor Simvastatin, reversed the resistance to ADT and blocked bone metastasis in CRPC cells. Finally, high MYBL2 levels were positively associated with TNM stage, total PSA level, and Gleason score and predicted a higher risk of metastatic relapse and poor prognosis in patients with PCa.

**Conclusions:** Our results reveal a novel molecular mechanism conferring resistance to ADT and provide a strong rationale for potential therapeutic strategies against CRPC.

## Introduction

Prostate cancer (PCa) is the second most frequent cancer and the fifth leading cause of cancer-related deaths in men 1. Androgen-deprivation therapy (ADT) is the mainstay treatment for newly diagnosed advanced PCa and recurrence after radical therapy, owing to the lineage-specific dependence on androgen receptor (AR) signaling at all stages of PCa progression 2. ADT initially results in tumor remission, but subsequently elicits resistance, ultimately leading to relapse with a more aggressive and often metastatic disease, termed castration-resistant prostate cancer (CRPC) 3. Patients with CRPC have poor clinical outcomes, with a median survival of 9-30 months 4. Current treatments for metastatic CRPC that target the androgen receptor (AR) axis such as enzalutamide and abiraterone improve patient survival but ultimately fail 5. However, growing evidence suggests that stimulation of alternative oncogenic signaling programs to bypass androgen dependency plays a critical role in CRPC development.

The Hippo-YAP pathway was initially identified as an evolutionarily conserved regulator of tissue growth and has been shown to control tumorigenesis, metastasis, and chemotherapy resistance in various human cancers 6, 7. Previous studies have demonstrated that Hippo-YAP signaling plays a crucial role in CRPC development. In primary tumors, nuclear expression of YAP has been shown to be significantly associated with tumor recurrence after primary treatment 8, while YAP has also been shown to be upregulated and markedly hyperactivated in castration-resistant prostate tumors compared with primary tumors 9. Moreover, ectopic expression of YAP facilitates the progression of androgen-sensitive LNCaP PCa cells to an androgen-independent status* in vitro* and confers castration-resistant growth of PCa cells* in vivo* 9. Importantly, inhibition of YAP activity *in vivo* has been shown to prevent PCa recurrence in castrated mice 8. Nonetheless, the precise mechanism that leads to dysregulation of the Hippo-YAP pathway during PCa progression to castration resistance is unknown.

MYB proto-oncogene like 2 (MYBL2) belongs to the MYB transcription factor family and plays an important role in regulating cell cycle progression, cell survival, and apoptosis 10-12. MYBL2 is widely expressed in proliferating cells and is required for inner cell mass formation during early embryonic development 13, 14. Overexpression of MYBL2 has been observed in various types of cancer and has been linked to aggressive tumor growth and poor clinical prognosis 15-17. Interestingly, microarray-based bioinformatic analyses revealed that MYBL2 was significantly overexpressed in prostate metastases and WISH-PC14 xenografts, derived from an androgen-insensitive metastatic prostate tumor 18. Furthermore, constitutive expression of MYBL2 allowed BALB/c 3T3 fibroblasts to grow with reduced growth factor requirements 19. Knockdown of MYBL2 significantly inhibited the proliferation of 22RV1 cells in the absence of androgens 20, implying the involvement of MYBL2 in metastatic CRPC development. Notably, CRPC-related YAP cooperates with the Myb-MuvB (MMB) complex subunit of MYBL2 to induce mitosis-associated gene expression and to support the proliferation of lung and liver cancer cells 21, 22. However, the clinical significance and biological functions of MYBL2 in advanced PCa progression and the cross-talk between MYBL2 and YAP in prostate tumor cells require further investigation.

In the present study, we found that MYBL2 was significantly upregulated in CRPC samples. Overexpression of MYBL2 inhibited Hippo signaling and stimulated YAP activity by inducing Rac GTPase activating protein 1 (RACGAP1)-mediated RhoA activation and conferred ADT resistance and a metastatic phenotype in androgen-dependent PCa cells. Silencing MYBL2, or treatment with either the YAP/TAZ inhibitor Verteporfin or the RhoA inhibitor Simvastatin, notably blocked PCa castration-resistant growth and bone metastasis in a castrated mouse model. These results demonstrate that MYBL2 functions as a key player in CRPC progression by acting upstream of RACGAP1 and uncoveres a novel mechanism for constitutive YAP activation in PCa.

## Materials and Methods

### Cell lines and cell culture

The human PCa cell lines, LNCaP, 22Rv1, VCaP, PC-3, MDA PCa 2b, and C4-2B, and the immortalized prostate epithelial cell lines, RWPE-1 and HPrEC, were purchased from the American Type Culture Collection (ATCC). LNCaP and 22Rv1 cells were cultured in RPMI-1640 medium (Invitrogen) supplemented with 10% fetal bovine serum (FBS, HyClone). VCaP cells were grown in Dulbecco's modified Eagle's medium (DMEM, Invitrogen) supplemented with 10% FBS. PC-3 and MDA PCa 2b cells were cultured in F-12K medium (Invitrogen) supplemented with 10% FBS. C4-2B cells were maintained in T-medium (Invitrogen) supplemented with 10% FBS. RWPE-1 and HPrEC cells were cultured in Keratinocyte Serum Free Medium (K-SFM) kit (Invitrogen). All cell lines were authenticated by short tandem repeat (STR) profiling. The cells were grown in a humidified incubator under 5% CO_2_ at 37 °C. All cell lines were tested regularly for mycoplasma using the LookOut Mycoplasma PCR Detection Kit (Sigma-Aldrich).

### Establishment of the androgen-independent LNCaP (LNCaP-AI) subline

The LNCaP-AI subline was constructed as described previously 23. Briefly, the parental LNCaP cells were cultured in RPMI-1640 supplemented with 10% charcoal-stripped FBS (CS-FBS; Gibco). The steroid-free culture medium was replaced two or three times a week, and the cells were passaged after dissociation at 1:2 or 1:4 split ratios approximately once a week. After approximately 6 months, a derivative of the LNCaP cells that was adapted to steroid-deprived conditions (LNCaP-AI cells) was achieved and maintained in RPMI-1640 supplemented with 10% CS-FBS.

### Patients and tissue specimens

This study was conducted on 187 archived paraffin-embedded clinical specimens (including 21 benign prostatic tissues, 132 primary or localized PCa tissues, and 34 CRPC specimens), which were both clinically and pathologically diagnosed. These tissues were obtained during surgery or needle biopsy at the First Affiliated Hospital of Sun Yat-sen University between January 2010 and December 2017. Prior informed consent from the patients and ethics approval from the Institutional Research Ethics Committee were obtained for the use of clinical samples for research purposes. This study was conducted in accordance with the 1975 Declaration of Helsinki.

### Plasmid construction, transfection, and establishment of stable cell lines

Full-length cDNA encoding human MYBL2 was PCR-amplified and cloned into a pMSCV-puro-retro vector (TaKaRa). To silence endogenous MYBL2, two short hairpin RNA (shRNA) oligonucleotide sequences targeting human MYBL2 were constructed into the pLKO.1-puro vector (Invitrogen). Different regions of the human MYBL2 promoter sequences generated by PCR amplification were cloned into the pGL3 luciferase reporter plasmid (Promega) to construct the corresponding luciferase reporters. A site-specific mutagenesis kit (Stratagene) was used to synthesize promoter reporter constructs with mutations in the MYBL2-binding motif. Transfection of plasmids was performed using Lipofectamine 3000 (Invitrogen) according to the manufacturer's protocol. Stable cell lines expressing MYBL2 or MYBL2-shRNA were generated by retroviral or lentiviral infection, respectively, and selected with 0.5 μg/mL puromycin after a 10-day culture period. Cells infected with the pGL3 luciferase retrovirus were selected with 250 μg/mL G418.

### Western blotting analysis

Cells were harvested in lysis buffer [50 mmol/L Tris (pH 6.8), 1% SDS, 10% Glycerol, and protease inhibitor cocktail (Sigma)] and heated for 5 min at 100 °C. The protein concentration was measured using the bicinchoninic acid (BCA) assay (Pierce) following the manufacturer's instructions. Equal quantities of protein were separated electrophoretically on 9% SDS/polyacrylamide gels and transferred onto polyvinylidene difluoride membranes (Roche). The membranes were probed with a primary antibody. Protein expression was determined using a horseradish peroxidase-conjugated secondary antibody and enhanced chemiluminescence (Pierce) according to the manufacturer's suggested protocols. The following primary antibodies were used in this study: anti-MYBL2 rabbit antibody (#PA5-79713; Invitrogen), anti-RACGAP1 rabbit antibody (#NBP1-33455; Novus), anti-YAP mouse antibody (#12395; Cell Signaling Technology), anti-p-YAP (S127) rabbit antibody (#13008; Cell Signaling Technology), anti-TAZ mouse antibody (#71192; Cell Signaling Technology), anti-LATS1 rabbit antibody (#3477; Cell Signaling Technology), anti-p-LATS1 (T1079) rabbit antibody (#8654; Cell Signaling Technology), anti-RhoA mouse antibody (ab54835; Abcam), anti-ECT2 rabbit antibody (07-1364; Sigma-Aldrich), anti-c-PARP mouse antibody (#32563; Cell Signaling Technology), and anti-c-Caspase3 rabbit antibody (#9661; Cell Signaling Technology). Goat anti-rabbit immunoglobulin G (ab7090; Abcam) and goat anti-mouse immunoglobulin G (ab97040; Abcam) were used as secondary antibodies. The membranes were stripped and re-probed with an anti-α-tubulin antibody (T9026; Sigma-Aldrich) as a loading control. P84 (ab487; Abcam) was used as a nuclear marker. For the RhoA activity assay, cells were lysed and immunoprecipitated with anti-active RhoA antibody (#NB-26904, NewEast Biosciences) and then immunoblotted with anti-RhoA antibody (ab54835; Abcam).

### RNA extraction, reverse transcription, and quantitative real-time PCR (qRT-PCR)

Total RNA was extracted from the indicated PCa cells and tissues using Trizol reagent (Invitrogen) and reverse-transcribed to cDNA using M-MLV Reverse Transcriptase (Promega) according to the manufacturer's instructions. qRT-PCR analysis was conducted using TB Green Fast qPCR Mix (Takara) on a CFX96 Real-Time System C1000 Cycler (Bio-Rad Laboratories). Human mRNA expression data were normalized to the housekeeping gene, GAPDH. The relative expression levels of the target genes were calculated as 2^-[(Ct of gene) - (Ct of GAPDH)]^, where Ct represents the threshold cycle for each transcript. The designed qRT-PCR primers are listed in Table S1.

### Immunofluorescence staining

PCa cells (2 × 10^4^) were cultured on coverslips in a 24-well plate and grown to 70% confluence. The cells were then washed with phosphate buffered saline (PBS) and fixed with 4% paraformaldehyde. After rinsing with PBS, the cells were blocked with PBS containing 1% Triton X-100. Following incubation with anti-YAP rabbit antibody (Alexa Fluor® 647 Conjugate, #38707; Cell Signaling Technology) at 4 °C overnight, the cells were stained with antifade reagent with DAPI (#8961, Cell Signaling Technology) to visualize the nuclei. Finally, the cells were rinsed in PBS and examined using a confocal laser scanning microscope system Olympus FV1000 (Olympus Medical Systems, Tokyo, Japan).

### Immunohistochemistry (IHC)

IHC staining was performed on paraffin-embedded tissue sections using the Histostain-Plus Kit (ThermoFisher) according to the manufacturer's instructions, as described previously 24. The primary antibodies used for IHC staining included anti-MYBL2 rabbit antibody (#PA5-79713; Invitrogen), anti-RACGAP1 rabbit antibody (#NBP1-33455; Novus), and anti-YAP mouse antibody (#12395; Cell Signaling Technology). Immunostaining was evaluated and scored separately by two independent pathologists who were blinded to the clinical data of the patients. Tumor cell proportions were scored according to the following criteria: 0, no positive tumor cells; 1, < 10% positive tumor cells; 2, 10%-35% positive tumor cells; 3, 35%-75% positive tumor cells; and 4, > 75% positive tumor cells. The protein staining intensity was graded using the following criteria: absent (no staining), scored as 0; weak staining (light yellow staining), scored as 1; moderate staining (yellow brown staining), scored as 2; and strong staining (brown staining), scored as 3. Protein expression was determined by calculating the staining index (SI) as the sum of the staining intensity score multiplied by the proportion of positive tumor cells, with possible scores of 0, 1, 2, 3, 4, 6, 8, 9, and 12. All scores were subdivided into two categories: specimens with an SI ≥ 6 were defined as having high expression, and those with an SI < 6 were defined as having low expression, according to the optimal threshold value of the receiver operating characteristic (ROC) curve.

### Cell viability assay

For the cell viability assay, 2.0 × 10^3^ PCa cells were plated per well in 96-well plates. The proliferation rate was measured by the WST-1 assay using the WST-1 Cell Proliferation and Cytotoxicity Assay Kit (Beyotime, Shanghai, China). Briefly, the WST-1 assay reagent was added to the cell culture media and incubated for 1 h. Then, the plate was shaken to mix the contents for 1 min, and the amount of formazan dye produced was determined by measuring the absorbance at 450 nm.

### Colony formation assay

The colony formation assay was performed as previously described 25. Briefly, 1 × 10^3^ PCa cells per well were seeded into 6-well plates. After 10-14 days in culture, colonies were fixed with methanol, stained with crystal violet, and counted under microscopy. For quantification, survival colonies formed by more than 50 cells were counted, and the survival fractions were calculated.

### Terminal deoxynucleotidyl transferase nick-end labeling (TUNEL) assay

Apoptosis in tissues was detected using a TUNEL staining kit (KeyGen Biotech) according to the manufacturer's protocol. Briefly, tissue sections were deparaffinized, rehydrated, and permeabilized. After incubation in TdT Enzyme reaction mix, the tissues were labeled with Streptavidin-TRITC at 37 °C in a humidified box for 30 min. The tissue sections were counterstained with DAPI, and the localized red fluorescence indicating apoptotic areas was detected by fluorescence microscopy. In each case, the percentage of apoptotic cells in 500 cells per field was microscopically counted and used to evaluate the mean apoptotic index.

### Apoptosis assay

Apoptosis assays were employed to evaluate cell apoptosis using the Annexin V-FITC/PI Apoptosis Detection Kit (KeyGEN BioTECH) according to the manufacturer's instructions. Briefly, 2 × 10^6^ cells were collected by centrifugation, resuspended in binding buffer, stained with fluorochrome-conjugated Annexin V and propidium iodide, and quantified by flow cytometry (Beckman Coulter, Brea, CA, USA).

### Luciferase activity assay

The luciferase activity assay was initiated by plating the indicated cells (3 × 10^3^ cells/well) in 48-well plates in triplicate. After 24 h in culture, 100 ng of the indicated luciferase reporter plasmids or the control luciferase plasmid, plus 3 ng pRL-TK Renilla plasmid (Promega), was transfected into the PCa cells using Lipofectamine 3000 (Invitrogen) following the manufacturer's protocol. Luciferase and Renilla signals were detected 24 h after transfection using the Dual Luciferase Reporter Assay Kit (Promega) according to the manufacturer's recommendations.

### Chromatin immunoprecipitation (ChIP) assay

The ChIP assay was conducted using the SimpleChIP Enzymatic Chromatin IP Kit (Magnetic Beads) (Cell Signaling Technology) following the manufacturer's protocol, as described previously 26. Briefly, the indicated cells (4 × 10^6^) were plated in a 100-mm culture dish and treated with 1% formalin to cross-link the proteins to DNA. Glycine (1 ×) was used to terminate the cross-linking. The indicated cells were lysed in SDS buffer, and sonication was used to fragment the DNA (ranging from to 300-1,000 bp). Sheared chromatin (10 μg) was incubated with 5 μg of anti-MYBL2 antibody (sc-390198 X; Santa Cruz Biotechnology), anti-H3K4me3 (#9751; Cell Signaling Technology), anti-p300 (#ab14984; Abcam), anti-RNA polymerase II (#05-623; Millipore), or anti-immunoglobulin G antibody (#I8765; Sigma-Aldrich) overnight at 4 °C with constant rotation. ChIP-grade protein G magnetic beads were added and incubated for 2 h at 4 °C with rotation. The immunoprecipitated chromatin was rinsed with low- and high-salt ChIP buffer. PCR was performed after releasing the DNA fragments from the protein/DNA cross-linking. The ChIP efficiency of certain binding sites was measured using the percentage of chipped DNA against input chromatin. The primers used for the ChIP assay are listed in Table S2.

### *In vivo* animal studies

All animal experiments were approved by the Institutional Animal Care and Use Ethics Committee of Sun Yat-sen University, and the experimental procedures were performed in accordance with the institutional guidelines. Male BALB/c nude mice (5 weeks old, 18-20 g) were purchased from the Slac-Jingda Animal Laboratory (Hunan, China) and raised in a barrier facility under a 12-h light/dark cycle. The mice were anesthetized using isoflurane inhalation. For the intracardiac injection model of bone metastasis, PCa cells (5 × 10^5^) suspended in 100 μL of PBS solution were injected into the left ventricle of anesthetized mice using an insulin syringe (28.5-gauge, 300 μL). Bone metastasis in the mice was detected twice a week using the IVIS Spectrum Imaging System (Caliper Life Sciences, Hopkinton, MA, USA). The incidence of bone metastasis was evaluated based on the luminescent signals in the mouse bones. The area of the osteolytic lesions caused by tumor metastasis in the two hind limbs was quantified using the Inveon Micro-CT/PET system (Siemens, Erlangen, Germany) and was expressed in square millimeters. Mice were euthanized on the indicated day. The two hind limbs were excised, fixed in formalin, decalcified for 3 weeks, embedded in paraffin, and subjected to hematoxylin and eosin (H&E) staining. Histomorphometric analysis was performed using Image-Pro Plus 7.0 software (Media Cybernetics, MD, USA), and the tumor burden of individual mice was defined as the area of bone occupied by tumor cells as validated by H&E staining.

To investigate whether MYBL2 overexpression confers castration-resistant growth *in vivo*, 5 × 10^6^ LNCaP-AI cells suspended in 100 mL PBS with 50% Matrigel (BD Biosciences) were injected subcutaneously into the flank region of intact BALB/c-nude mice (n = 8/group). Surgical castration was performed when the tumor xenografts reached a volume of approximately 200 mm^3^. The tumor volume was measured using an external caliper and calculated using the formula (L × W^2^)/2, where L is the tumor length and W is the width. The kinetics of tumor formation and growth were measured by monitoring the tumor volume every 3 days. After the mice were sacrificed, the tumors were excised and weighed. The tumors were then dissected and subjected to IHC analysis. The proliferative index was scored by calculating the percentage of Ki67-positive cells. The apoptotic index based on TUNEL staining (DeadEnd™ Fluorometric TUNEL System, Promega) was determined by counting the percentage of TUNEL-positive cells.

To evaluate the effect of Verteporfin (Sigma-Aldrich) or Simvastatin (Sigma-Aldrich) on castration-resistant growth and bone metastasis *in vivo*, tumor-bearing mice were randomly grouped and treated with Verteporfin (100 mg/kg body weight, every 2 days, intraperitoneal injection) or Simvastatin (5 mg/kg body weight, 5 days a week, intraperitoneal injection) alone.

### Bioinformatics and data analysis

MYBL2 mRNA levels in prostate tissues and PCa tissues were assessed by analyzing the GSE21032, GSE10645, and GSE35988 PCa mRNA datasets acquired from the Gene Expression Omnibus (GEO) (http://www.ncbi.nlm.nih.gov/geo/), and the prostate adenocarcinoma (PRAD) dataset acquired from The Cancer Genome Atlas (TCGA) data portal (https://tcga-data.nci.nih.gov/tcga/tcgaHome2.jsp). We downloaded the series matrix files of microarray datasets from the GEO and RNA-Seq data from the TCGA database. Gene expression was presented as the mean value of multiple probes for each gene after log2 transformation. Samples in the TCGA datasets were separated into high or low MYBL2 expression groups using the extreme quartiles, as previously described 27. Using this method, samples with the lowest quartiles were defined as MYBL2-low, and those with the highest quartiles were defined as MYBL2-high.

### Statistical analysis

Statistical analyses were conducted using SPSS 21.0 or GraphPad Prism 7 software. Student's t-test was used for comparisons between the two groups, and analysis of variance (ANOVA) was used to analyze the differences between more than two groups. The χ^2^ test was used to analyze the relationship between MYBL2 expression and clinicopathological characteristics. Survival curves were plotted using the Kaplan-Meier method and were compared using the log-rank test. Multivariate statistical analysis was performed using Cox proportional hazard regression models. Results were considered statistically significant at *P* < 0.05.

## Results

### MYBL2 shows upregulated expression in CRPC tissues and cell lines

**Figure 1:**
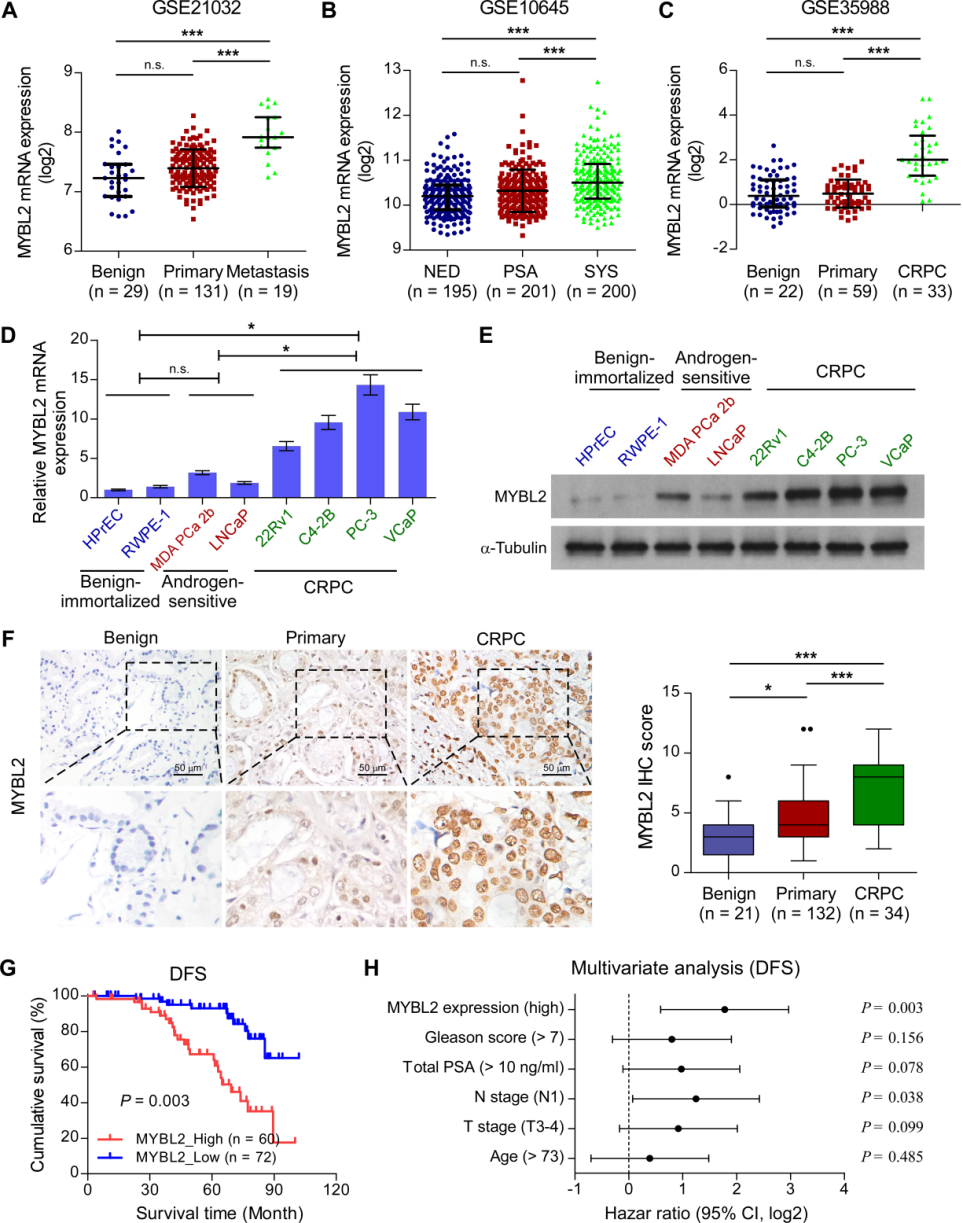
** Upregulation of MYBL2 contributes to the development of CRPC.** (A-C) MYBL2 mRNA levels in PCa tissues were assessed by analyzing the GSE21032 (A), GSE10645 (B), and GSE35988 (C) PCa mRNA datasets (NED control: No evidence of disease, PSA control: PSA recurrence but no evidence of clinical progression within 5 years, SYS: Systemic progression within 5 years after PSA recurrence, CRPC: Castration-resistant prostate cancer). (D) QRT-PCR analysis of MYBL2 expression in the indicated cell lines. GAPDH was used an internal control. In (A-D), *P*-values were determined by one-way ANOVA test. (E) Western blotting analysis of MYBL2 expression in the indicated cell lines. α-Tubulin was used as a loading control. (F) Left panel: Representative IHC images of MYBL2 in benign prostate tissues (n = 21), primary tissues (n = 132), and CRPC tissues (n = 34). Scale bars: 50 μm. Right panel: Statistical quantification of the MYBL2 IHC score (one-way ANOVA test). (G) Kaplan-Meier analysis of disease-free survival (DFS) curves for patients with PCa with low MYBL2 expression versus high MYBL2 expression (log-rank test, n = 132). (H) Multivariate analysis of DFS by Cox-regression analysis in patients with PCa (n = 132 cases). Data were presented as mean ± SD. *P < 0.05, ***P < 0.001, n.s, no significance.

To explore the role of MYBL2 in advanced PCa progression, we first analyzed the MYBL2 expression profile in clinical prostatic tumors using publicly available GEO and TCGA data. We found that MYBL2 mRNA levels were robustly upregulated in metastatic PCa samples compared with those in non-metastatic PCa or benign tissues, in T3-4 tumors compared with T2, in N1 PCa compared with N0, and correlated significantly with the Gleason score and PSA level (Figure 1A and Figure S1A-E). A similar increase in MYBL2 expression was observed in PCa tissues from patients with local or metastatic recurrence after prostatectomy, as compared with that in tissues from patients with no evidence of disease progression (Figure 1B and Figure S1F). Notably, the analysis revealed a stepwise upregulation of MYBL2 toward aggressiveness, showing the lowest expression in benign tissues, followed by primary or localized PCa tissues, and finally, metastatic CRPC specimens (Figure 1C). We further validated the data in the public domain. As shown in Figure 1D-E, both the mRNA and protein levels of MYBL2 were significantly higher in CRPC cell lines (22Rv1, C4-2B, PC-3, and VCaP) than in hormone-sensitive PCa cell lines (MDA PCa 2b and LNCaP) and in non-neoplastic prostate epithelial cells (HPrEC and RWPE-1). Consistently, IHC staining verified that the nuclear staining of MYBL2 was significantly stronger in patients with CRPC, while MYBL2 staining was weak in localized PCa and undetectable in benign prostate tissues (Figure 1F). These results suggest that MYBL2 is upregulated in CRPC tumors.

### Upregulation of MYBL2 correlates with advanced progression and poor prognosis in PCa

We then investigated whether the expression of MYBL2 was related to the clinicopathological characteristics of patients with PCa. As shown in Table S3, high expression of MYBL2 correlated significantly with T stage (*P* < 0.001), lymph node metastasis (*P* < 0.001), high total PSA level (*P* = 0.028), Gleason score (*P* = 0.045), and relapse or metastasis status in patients with PCa (*P* < 0.001). Notably, Kaplan-Meier survival curves and log-rank tests revealed that patients with high MYBL2 expression had significantly poorer disease-free survival (DFS) than those with low MYBL2 expression (*P* = 0.003; Figure 1G), which was consistent with the results from the TCGA PRAD cohort (Figure S1G). Additionally, multivariate analyses revealed that high MYBL2 expression and N stage were independent prognostic factors for DFS in patients with PCa (Figure 1H and Table S4). These findings implicate MYBL2 as a clinically significant player in the advanced progression of PCa and suggest that MYBL2 could be a marker of poor prognosis in PCa.

### MYBL2 confers resistance to androgen-deprivation treatment in PCa cells* in vitro*

**Figure 2:**
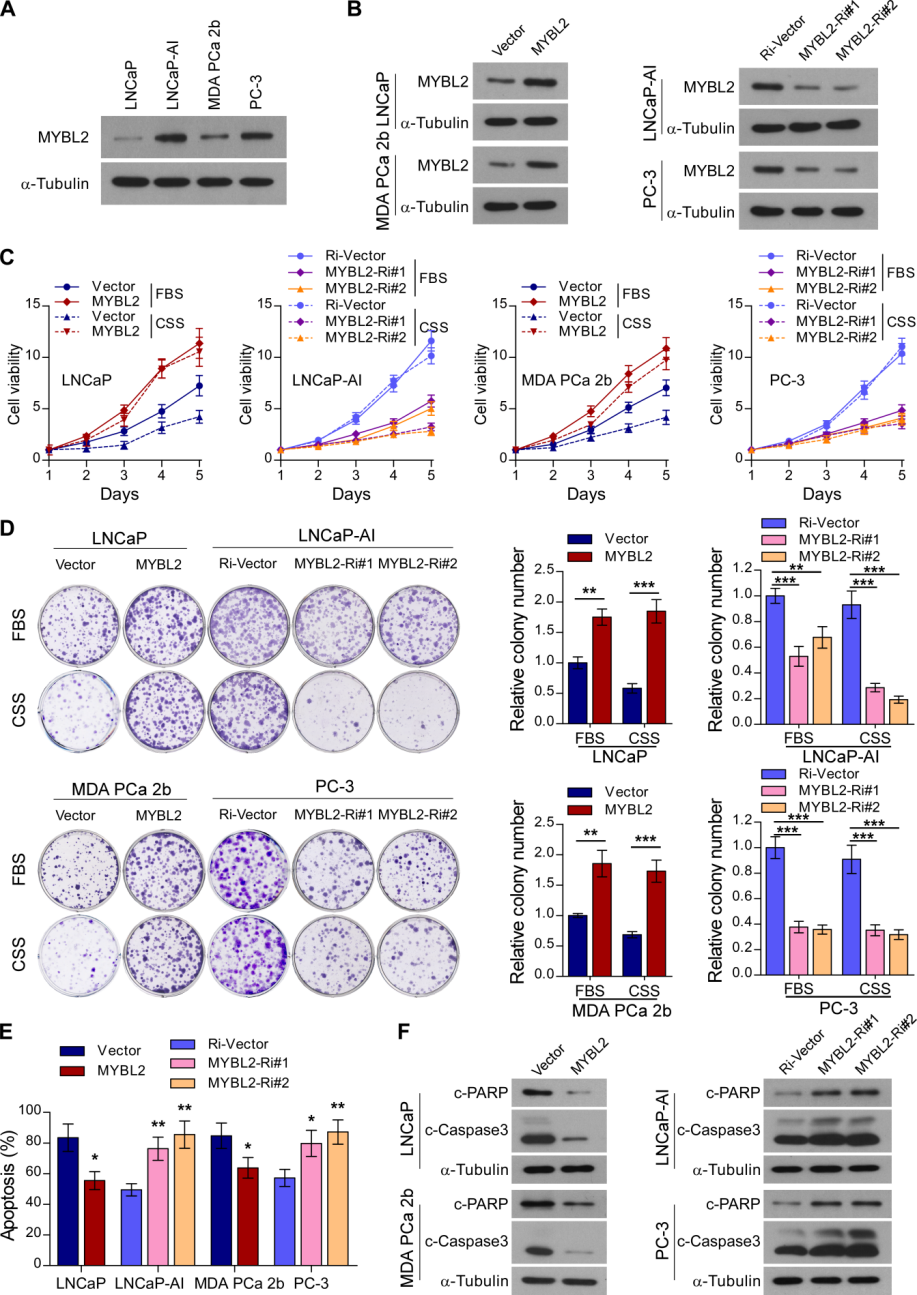
** MYBL2 confers resistance to androgen-deprivation treatment in PCa cells* in vitro*.** (A and B) Western blotting analysis of MYBL2 expression in the indicated cell lines. α-Tubulin was used as a loading control. (C) Cell viability was assessed in the indicated cells, cultured either in medium with fetal bovine serum (FBS) or with charcoal-stripped serum (CSS), every day for 5 days after seeding (two-way ANOVA test). (D) Representative images (left panel) and quantification (right panel) of colonies formed by the indicated cell lines cultured either in medium with FBS or CSS. (E) Apoptosis assessed by flow cytometry using Annexin V-FITC/PI staining in the indicated cells treated in medium supplemented with 0.5% CSS for 4 days. In (D-E), *P*-values were determined by two-tailed student's t test when comparing two groups and one-way ANOVA test when comparing three groups. (F) Western blotting analysis of the indicated protein expression in the indicated cells cultured in medium with CSS. α-tubulin was used as a loading control. Data were presented as mean ± SD, **P < 0.01, ***P < 0.001.

Next, we constructed an androgen-independent LNCaP-AI cell line derived from androgen-sensitive parental LNCaP cells by long-term culture in RPMI-1640 medium with charcoal-stripped serum (CSS). The MYBL2 expression level was significantly increased in androgen-independent LNCaP-AI and PC-3 cell lines compared to that in androgen-sensitive LNCaP cells and MDA PCa 2b cells (Figure 2A). To explore the biological function of MYBL2 and investigate whether it plays a positive role in CRPC progression, we established LNCaP and MDA PCa 2b, stably expressing MYBL2, as well as LNCaP-AI and PC-3 cell lines, stably transduced with an MYBL2 shRNA (Figure 2B).

Notably, MYBL2 overexpression dramatically accelerated the growth of LNCaP and MDA PCa 2b cells, while MYBL2 silencing markedly inhibited the growth rate of LNCaP-AI cells; the pro-proliferation function of MYBL2 on PCa cells was more remarkable in the castrated condition with CSS medium (Figure 2C). The proliferation of AR-negative PC-3 cells was significantly suppressed by MYBL2 silencing; however, the inhibitory effect was independent of the culture conditions (Figure 2C). Moreover, MYBL2-overexpressing PCa cells formed more and larger colonies than did the vector control, whereas colonies derived from MYBL2-knockdown androgen-independent PCa cells were significantly fewer and smaller, and an additional pro-growth effect of MYBL2 was evident under CSS conditions in all PCa cells, except PC-3 (Figure 2D). In addition, overexpression of MYBL2 reduced, while silencing of MYBL2 dramatically increased the percentage of apoptotic cells under androgen-ablated conditions, as well as the protein levels of classic apoptosis markers such as c-Caspase3 and c-PARP (Figure 2E-F). These results suggest that MYBL2 overexpression confers *in vitro* resistance to androgen-deprivation conditions in PCa cells.

### Silencing of MYBL2 suppresses castration-resistant growth and the bone metastatic capacity of CRPC cells

**Figure 3:**
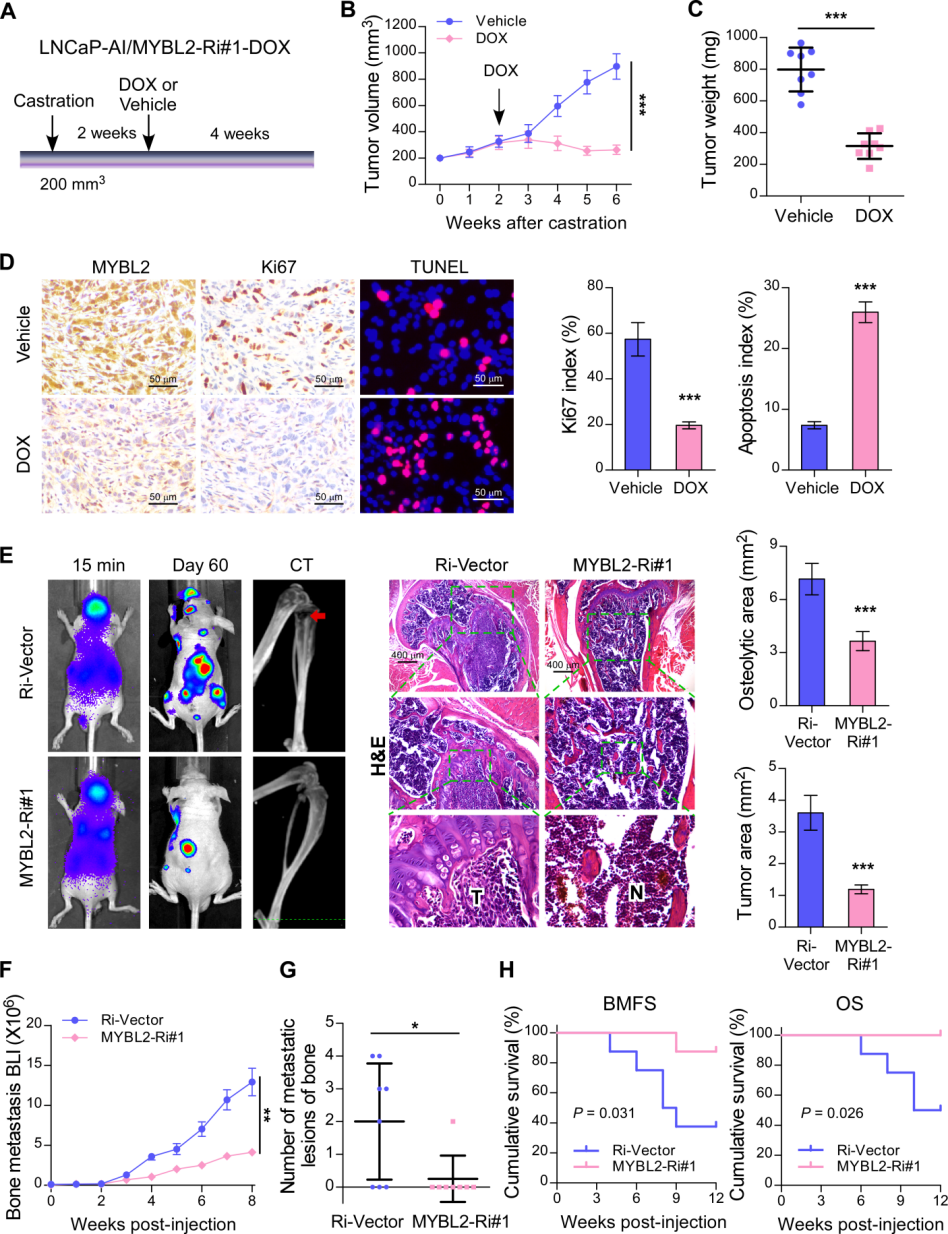
** Silencing of MYBL2 suppresses castration-resistant growth and bone metastatic capacity of CRPC cells.** (A) Schematic illustration of *in vivo* models of castration therapy performed in the mice bearing tumor xenografts formed by LNCaP-AI/MYBL2-Ri#1-DOX cells, followed by treatment with vehicle or doxycycline (DOX). (B) Tumor growth curves of the indicated xenograft tumors (n = 8/group) during the experiments shown in (A) (two-way ANOVA test). (C) Quantification of the xenograft tumor weight at the end of the experiments shown in (A). (D) Representative images (left panel) and quantification (right panel) of IHC staining of MYBL2 and Ki67, and TUNEL analysis of apoptotic cells in the xenografts tissues. Scale bars: 50 μm. (E) Representative BLI, Micro-CT, and histological images (H&E) of bone lesions from mice inoculated intracardially with PC-3/Ri-vector or PC-3/MYBL2-Ri#1 cells in each experimental group (n = 8/group). T, tumor tissues; N, normal tissues. Scale bars: 400 μm. Quantification of hind-limb osteolysis in each experimental group using Micro-CT analysis (right upper panel). Histomorphometric quantification of the tumor area in hind limbs from each experimental group (right lower panel). In (C-E), *P*-values were determined by two-tailed student's t test. (F) The growth curve for bone metastasis burden as quantified by BLI in each group (two-way ANOVA test). (G) Numbers of metastatic lesions in bones from each mouse in two groups (Student's t test). (H) Kaplan-Meier curves of bone metastasis-free survival (BMFS) and overall survival (OS) in mice inoculated with PC-3/Ri-vector or PC-3/MYBL2-silenced cells (log-rank test). Data were presented as mean ± SD. *P < 0.05, ***P < 0.001.

To investigate whether silencing of MYBL2 could improve the anti-tumor effect of ADT treatment in established CRPC tumors* in vivo*, an LNCaP-AI cell line with a doxycycline-inducible MYBL2 shRNA (MYBL2-Ri#1-Dox) was constructed. LNCaP-AI/MYBL2-Ri#1-Dox cells mixed with Matrigel were subcutaneously injected into the inguinal folds of BALB/c nude mice. Castration treatment was initiated once the tumor volume reached approximately 200 mm^3^, and the doxycycline regimen or vehicle was introduced approximately 2 weeks later (Figure 3A). The *in vivo* animal model results supported that doxycycline treatment significantly blocked LNCaP-AI/MYBL2-Ri#1-Dox xenograft tumor growth in castrated mice (Figure 3B-C). Importantly, doxycycline treatment resulted in reduced expression of Ki67 and an increased percentage of apoptotic cells in LNCaP-AI/MYBL2-Ri#1-Dox tumor xenografts compared with that in the vehicle-treated groups (Figure 3D). These results suggest that MYBL2 silencing inhibits castration-resistant growth of PCa cells *in vivo*.

CRPC represents an advanced stage of PCa that progresses despite ADT and correlates with frequent bone metastases 28. To study the role of MYBL2 in PCa bone metastasis directly *in vivo*, we established a rapid bone metastasis model using intracardiac injections of luciferase-labeled PC-3 cells in BALB/c-nude mice. Bone metastatic lesion areas in the MYBL2-silencing group were much smaller than those in the vector control group according to micro-CT and histomorphometric analyses (Figure 3E). The growth curve showed that silencing of MYBL2 significantly decreased castration-resistant tumor growth in the hindlimb bone microenvironment (Figure 3F). Importantly, MYBL2 knockdown significantly reduced the incidence of bone metastasis (from 62.5% to 12.5%) and the number of bone metastatic lesions (from 2 to 0.25) in tumor-bearing mice (Figure 3G). Repeated experiments and subsequent survival analysis revealed that MYBL2 silencing notably delayed the onset of bone metastasis and prolonged the overall survival of mice (Figure 3H). These findings suggest that MYBL2 plays a critical role in promoting the colonization and growth of PCa cells within the bone.

### MYBL2 inhibits Hippo signaling leading to YAP activation

**Figure 4:**
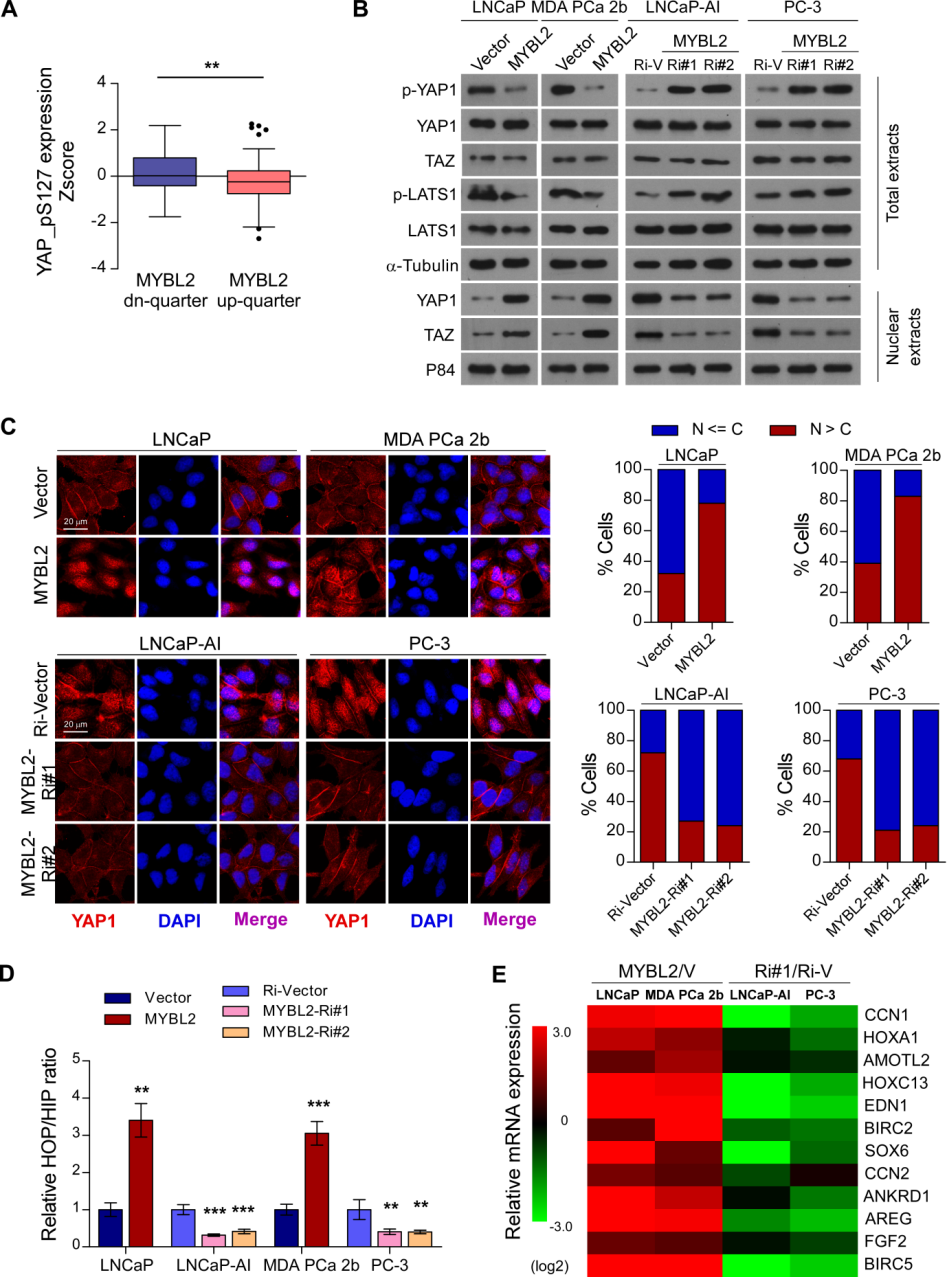
**MYBL2 inhibits Hippo signaling leading to YAP activation.** (A) Analysis of the TCGA PCa protein dataset revealed that p-YAP-S127 expression was significantly downregulated in PCa samples with the highest quartiles versus those with the lowest quartiles of MYBL2 (two-tailed Student's t test). (B) Western blotting analysis of the indicated protein expression in the indicated cells. α-tubulin was used as a loading control for total extracts; P84 was used as a loading control for nuclear extracts. (C) Left panel: Expression and subcellular localization of YAP1 were analyzed in the indicated cells with altered MYBL2 expression and visualized by fluorescence and laser confocal microscopy. Scale bars: 20 μm. Right panel: Percentage of subcellular localization of YAP1 in the indicated cells (Nucleus (N); Cytoplasm (C)). (D) HOP/HIP luciferase activity was analyzed in the indicated cells. HOP-Flash luciferase assay shows YAP/TAZ-TEAD transcriptional activity; HIP-Flash is a HOP-flash mutant.* P*-values were determined by two-tailed Student's t test when comparing two groups and one-way ANOVA test when comparing three groups. (E) Fold change of the mRNA expression of indicated genes in qRT-PCR analysis comparing cells overexpressing MYBL2 versus vector (V) or MYBL2-Ri#1 (Ri#1) versus Ri-vector (Ri-V), generated by log2 transformation. Data were presented as mean ± SD. *P < 0.05, **P < 0.01, ***P < 0.001.

Inhibition of Hippo signaling plays an important role in favoring the acquisition of CRPC features and contribute to PCa aggressiveness 9. To further explore the mechanism by which MYBL2 regulates castration-resistant growth and metastasis of PCa cells, we analyzed the changes in protein expression profiles of the Hippo signaling pathway in TCGA datasets. We found that the levels of p-YAP1-S127, a negative form of YAP1, were significantly downregulated in MYBL2-high expressing PCa tissues compared to those in MYBL2-low tissues (highest *vs*. lowest quartile, 0.1620 ± 0.09511 versus -0.2084 ± 0.1013, Z-score values, *P* = 0.008) (Figure 4A). Western blotting analysis confirmed that MYBL2 overexpression reduced the levels of phosphorylated YAP1 and LATS1 and enhanced the abundance and nuclear levels of YAP and TAZ, whereas MYBL2 silencing had the opposite effect (Figure 4B). Meanwhile, fluorescence immunostaining indicated that MYBL2 overexpression induced the nuclear translocation of YAP1 in PCa cells (Figure 4C).

YAP is a transcription cofactor that can shuttle from the cytoplasm to the nucleus, where it stimulates gene transcription together with TEA-domain family members (TEADs) and another cofactor, TAZ. We employed HOP/HIP flash luciferase assays to further investigate the effect of MYBL2 on YAP/TAZ-TEAD transcriptional activity. As expected, the activity ratio of HOP/HIP increased significantly in MYBL2-overexpressing androgen-dependent PCa cells and was reduced in MYBL2-knockdown CRPC cells (Figure 4D). Accordingly, the product of typical YAP/TAZ downstream target genes, such as CCN1, HOXA1, and AMOTL2, were upregulated significantly after ectopic expression of MYBL2, but were decreased in MYBL2 knockdown cells (Figure 4E). Notably, MYBL2 silencing-mediated suppression of YAP/TAZ-TEAD reporter activity, and typical YAP/TAZ downstream target gene expression was abrogated by LATS1 deletion (Figure S2A-B). In addition, the results of *in vitro* experiments indicated that silencing of LATS1 or transduction of mutant YAP-S127A (which cannot be inactivated by Hippo kinases) in PCa cells significantly reversed the anti-proliferation and pro-apoptotic effects after RNAi-mediated depletion of MYBL2 in androgen-ablated medium (Figure S2C-D). These results revealed that MYBL2 promotes and relies on YAP activity to induce castration resistance in PCa cells.

### MYBL2 transcriptionally upregulates RACGAP1, a critical repressor of Hippo signaling

**Figure 5:**
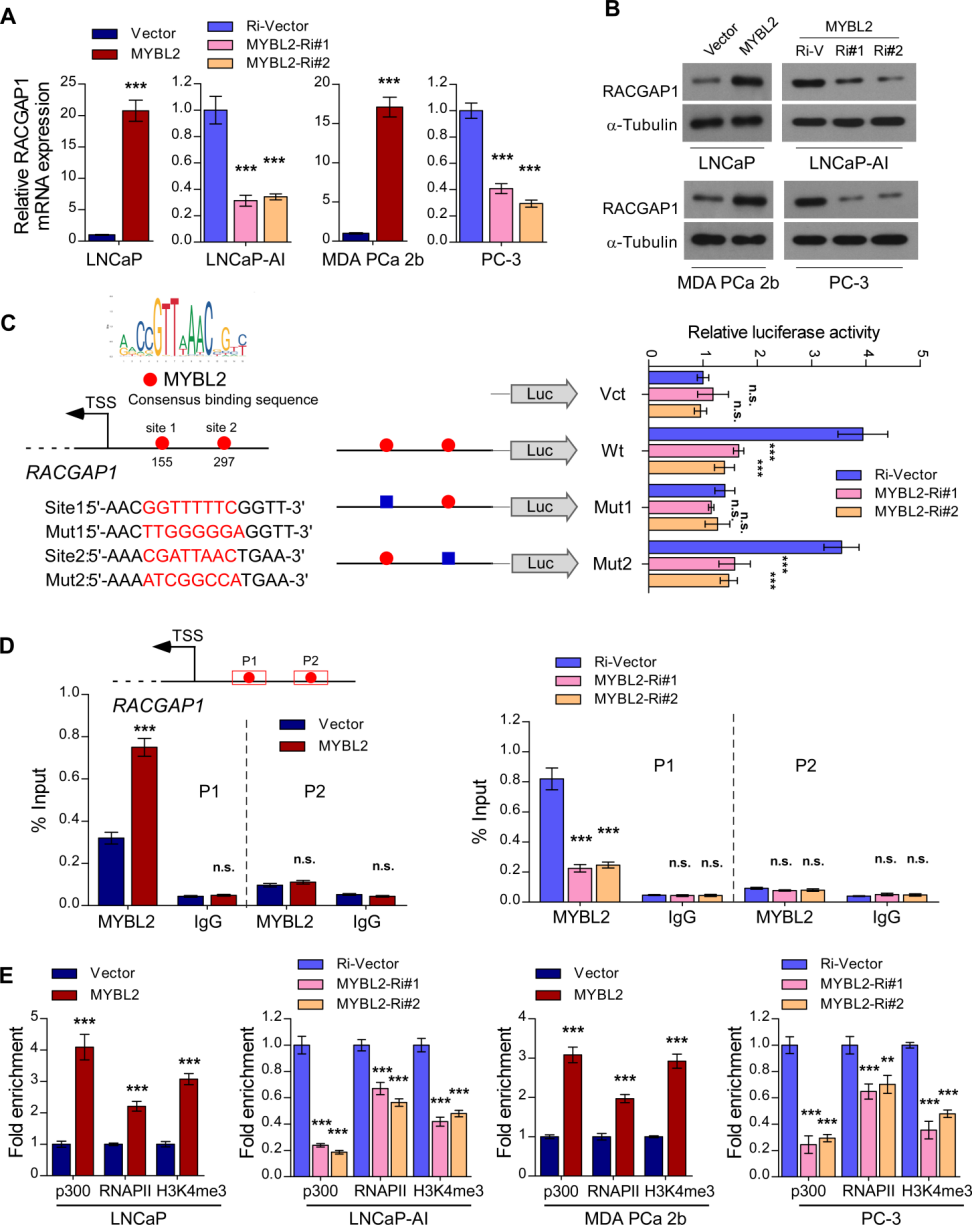
** MYBL2 transcriptionally upregulates RACGAP1, a critical repressor of Hippo signaling.** (A) QRT-PCR analysis of RACGAP1 expression in MYBL2 silenced-, MYBL2 overexpressing-, and control cells. GAPDH was used as an internal control. (B) Western blotting analysis of RACGAP1 expression in the indicated cells. α-Tubulin was used as a loading control. (C) Left upper panel: schematic illustration of the predicted binding site for MYBL2 in the indicated RACGAP1 promoter regions. Left lower panel: schematic illustration of the wild-type or mutant RACGAP1 promoter regions cloned into the pGL3 luciferase reporter plasmid. Right panel: quantification of luciferase activity of the RACGAP1 promoter reporter in the indicated cells. Putative MYBL2-binding sites are shown as red filled circles, and the blue filled box represents the mutated site. The red letters in each binding region indicate the putative or mutated MYBL2-binding sequences. Vct: Empty vector, Wt: Wild-type, Mut: Mutant. (D) ChIP analysis of enrichment of MYBL2 on the RACGAP1 gene promoter. IgG was used as a negative control. The red squares represent the qRT-PCR region. (E) ChIP assays were performed in the indicated cells using anti-p300 acetyltransferase, anti-RNA POL II (RNAP II), and anti-H3K4me3 antibodies. In (A, C-E), data were presented as mean ± SD, *P*-values were determined by two-tailed Student's t test when comparing two groups and one-way ANOVA test when comparing three groups. **P < 0.01, ***P < 0.001, n.s, no significance.

In the canonical Hippo signaling cascade, MST1/2 phosphorylates and activates LATS1/2, which in turn phosphorylates YAP1 at Ser127, leading to cytoplasmic retention and degradation of YAP1 29. As a member of the MYB family of transcription factors, MYBL2 localizes to the nucleus and exerts its effects by modulating downstream gene transcription. To explore the possible mechanism by which MYBL2 activates YAP/TAZ signaling, we examined whether MYBL2 regulates the transcription of negative modulators of Hippo signaling, such as NPHP4, TP53BP2, CIT, and RACGAP1 30-33. Interestingly, we observed a strong positive correlation between MYBL2 mRNA levels and RACGAP1 and CIT expression by analyzing the TCGA PRAD cohort (Figure S3A). qRT-PCR analysis confirmed higher RACGAP1 expression in LNCaP-AI cells than in parental LNCaP cells and significant downregulation in MYBL2-silenced PCa cells (Figure S3B-C), indicating that MYBL2 might modulate YAP activity by inducing RACGAP1 expression. Further qRT-PCR and western blotting analyses demonstrated that RACGAP1 was potently regulated by MYBL2 (Figure 5A-B). Moreover, we identified two putative MYBL2 binding sites on the promoter region of RACGAP1 using JASPAR 34. Luciferase reporter assays revealed that downregulation of MYBL2 attenuated the luciferase activity of the RACGAP1 promoter containing the first putative wild-type, but not mutated, MYBL2-binding site (Figure 5C). In addition, ChIP assays showed that endogenous MYBL2 could bind to the RACGAP1 promoter at the first binding site (Figure 5D). Furthermore, MYBL2 overexpression increased, while silencing of endogenous MYBL2 reduced the enrichment of p300, RNA polymerase II, and the gene-activating marks H3K4me3 on the RACGAP1 promoter (Figure 5E).

Importantly, the MYBL2-mediated increase in YAP/TAZ-TEAD reporter activity and typical YAP/TAZ downstream target gene expression was abrogated by RACGAP1-knockdown (Figure S3D-E). The results of *in vitro* experiments indicated that silencing RACGAP1 significantly reduced the colony number and increased the apoptotic rate of MYBL2-overexpressing PCa cells in androgen-ablated medium (Figure S3F-G). These results indicate that MYBL2 activates YAP1 signaling by enhancing RACGAP1 transcription, leading to PCa castration resistance.

### MYBL2 promotes YAP signaling by inducing RACGAP1-mediated RhoA activation

**Figure 6:**
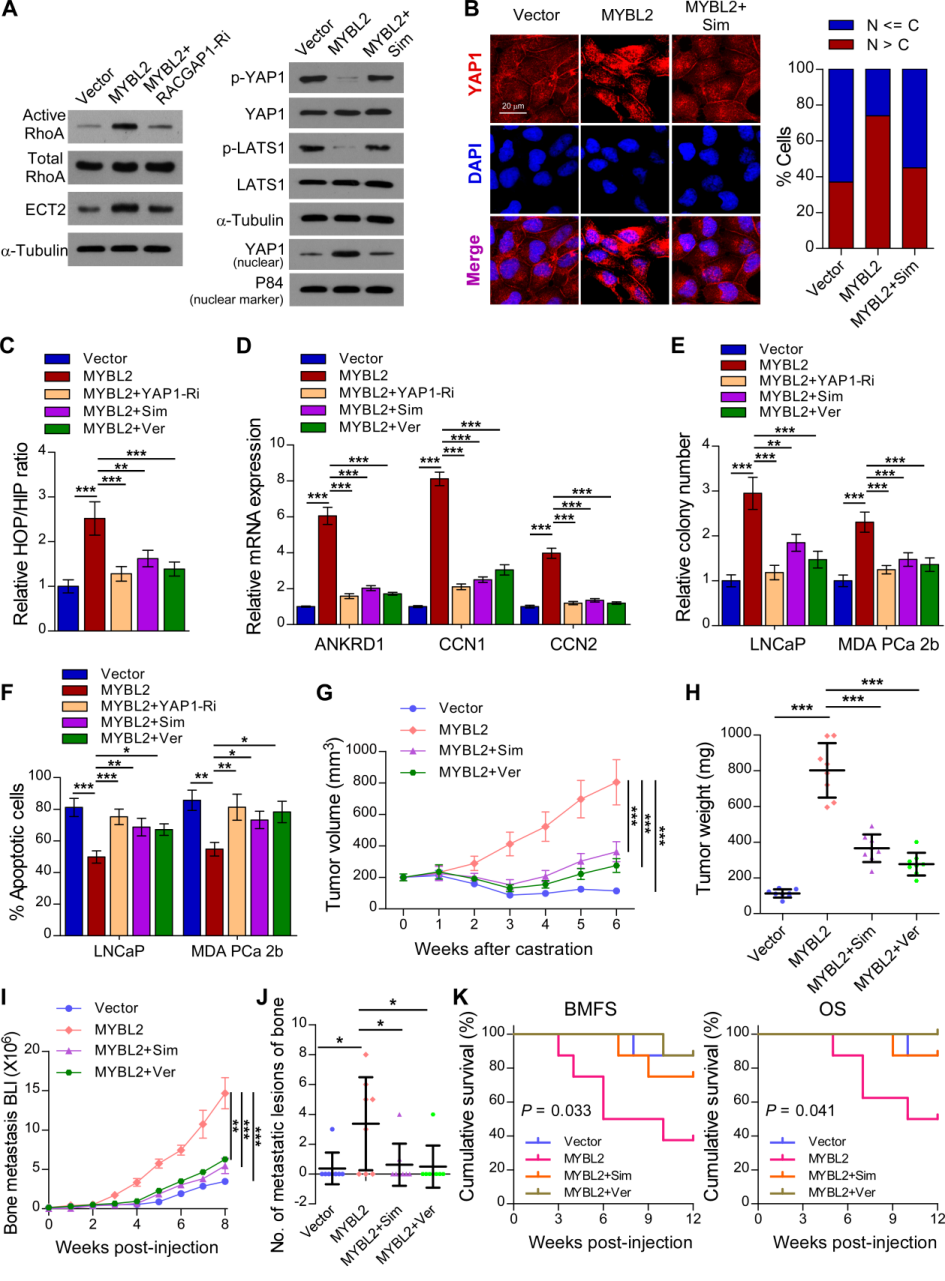
**MYBL2 promotes castration-resistance and bone-metastasis of PCa by activating RhoA and YAP signaling.** (A) For the RhoA activity assay, cells were immunoprecipitated with anti-active RhoA antibody and then immunoblotted with anti-RhoA antibody. A portion of the cell lysate (1/30) was also directly immunoblotted with anti-RhoA antibody. Western blotting analysis of the indicated protein expression in the indicated groups of LNCaP cells. α-tubulin was used as a loading control of total protein; P84 was used as a loading control of nuclear protein. Sim, Simvastatin, 5 μM, 24 h. (B) Left panel: Expression and subcellular localization of YAP1 was analyzed in the indicated groups of LNCaP cells with Simvastatin treatment and visualized by fluorescence and laser confocal microscopy. Scale bars: 20 μm. Right panel: Percentage of the subcellular localization of YAP1 in the indicated cells (Nucleus (N); Cytoplasm (C)). (C) Luciferase activity of the YAP/TAZ-TEAD promoter reporter was examined in the indicated groups of LNCaP cells. Simvastatin (5 μM, 24 h); Ver, Verteporfin (10 µM, 3 h). (D) QRT-PCR analysis of YAP1-targeted genes mRNA expression in the indicated groups of LNCaP cells. Gene expression levels were normalized to GAPDH. (E) Representative images (left panel) and quantification (right panel) of colonies formed by the indicated cells with Simvastatin or Verteporfin treatment. (F) Apoptosis assessed in the indicated cells treated in medium supplemented with 0.5% CSS for 4 days and subsequently treated with Simvastatin or Verteporfin. (G) Tumor growth curves of the indicated LNCaP xenograft tumors (n = 8/group). Mice were administered with intraperitoneal injection of Simvastatin (5 mg/kg, five days a week) or Verteporfin (100 mg/kg, every two days). (H) Quantification of xenograft tumor weight at the end of the experiment shown in (F). (I) Growth curve for bone metastasis burden as quantified by BLI in the indicated groups of MDA PCa 2b cells. (J) Numbers of metastatic lesions in bones from each group. (K) Kaplan-Meier curves of bone metastasis-free survival (BMFS) and overall survival (OS) in mice inoculated with the indicated groups of MDA PCa 2b cells (log-rank test). In (C-F, H and J), *P*-values were determined by one-way ANOVA test. In (G), *P*-values were determined by two-way ANOVA test. Data were presented as mean ± SD. *P < 0.05, **P < 0.01, ***P < 0.001.

A previous study demonstrated that RACGAP1 promotes ECT2-mediated RhoA activation, leading to F-actin formation and YAP activation 33. Meanwhile, RhoA promotes TAZ/YAP activation via both Hippo-LATS kinase cascade-dependent and -independent mechanisms in PCa cells 35. We further evaluated the importance of RACGAP1-mediated RhoA activation in MYBL2-induced YAP activation. As expected, the expression of active RhoA and ECT2 was upregulated in MYBL2-overexpressing PCa cells, while knockdown of RACGAP1 reversed these effects (Figure 6A). In addition, treatment with Simvastatin, a RhoA subfamily Rho GTPase inhibitor, significantly increased the levels of phosphorylated LATS1 and YAP1 and reduced the nuclear levels of YAP1 in MYBL2-overexpressing PCa cells (Figure 6A-B). Importantly, the MYBL2-mediated increase in YAP/TAZ-TEAD reporter activity and typical YAP/TAZ downstream target gene expression was reversed by Simvastatin, similar to the effects of YAP1 depletion or Verteporfin treatment (Figure 6C-D). These results suggest that RACGAP1-mediated RhoA activation is indispensable for MYBL2-induced YAP/TAZ activity.

### Simvastatin and Verteporfin abrogate the promotion of castration-resistant growth and bone metastasis by MYBL2

We next examined whether the pro-growth and pro-metastasis functions of MYBL2 are mediated by RACGAP1-mediated RhoA activation and YAP signaling activation. The results of an *in vitro* experiment indicated that either Simvastatin or Verteporfin treatment and YAP1 silencing significantly reduced the colony number and increased the apoptotic rate of MYBL2-overexpressing PCa cells in androgen-ablated medium (Figure 6E-F). In the *in vivo* experiments, the tumor xenografts formed by LNCaP-MYBL2 cells appeared to show no response to castration treatment and continued to grow aggressively at enhanced rates in contrast to tumor xenografts formed by LNCaP-Vector clones that stopped growing, and even shrank (Figure 6G-H). Importantly, administration of Simvastatin or Verteporfin resulted in persistent suppression of castration-resistant tumor growth in MYBL2 high-expressing LNCaP tumor xenografts (Figure 6G-H). In addition, the creation of a rapid bone metastasis model by intracardiac injections of MDA PCa 2b cells into nude mice revealed that MYBL2 overexpression increased the hindlimb tumor burden and the number of bone metastatic lesions, significantly accelerated the onset of bone metastasis, and markedly shortened the survival time in tumor-bearing mice (Figure 6I-K). Meanwhile, Simvastatin or Verteporfin almost completely reversed these effects (Figure 6I-K). These results indicate that MYBL2 relies on RACGAP1-mediated RhoA activation and YAP signaling to exert its castration-resistant and pro-metastatic effects on PCa cells.

### Clinical relevance of the MYBL2/RACGAP1/YAP axis in human PCa

**Figure 7:**
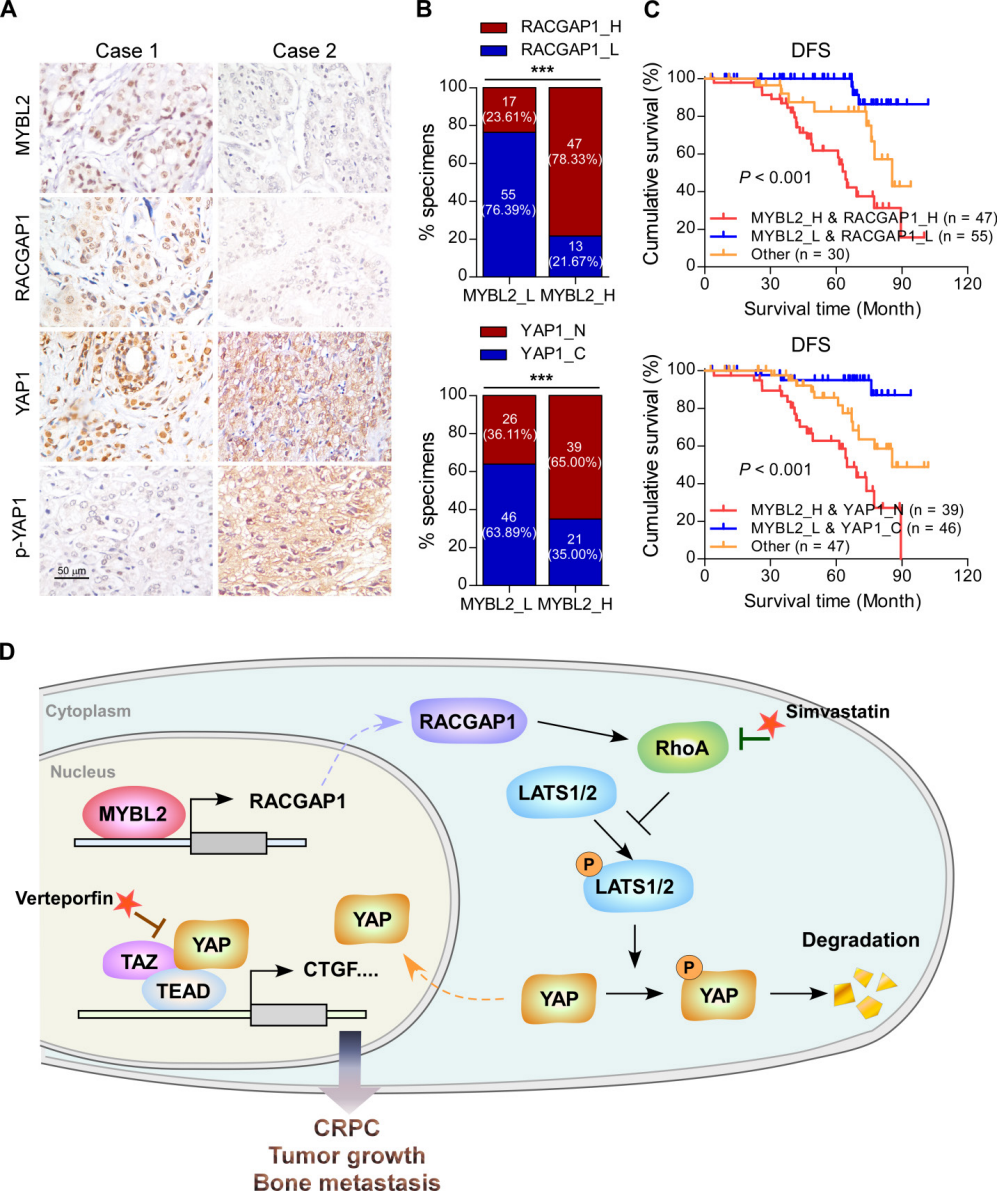
** Clinical relevance of the MYBL2/RACGAP1/YAP axis in human PCa.** (A) Representative images of MYBL2, RACGAP1, YAP1, and p-YAP1 IHC staining in 132 breast cancer patient specimens. Scale bars: 50 μm. (B) Percentage of PCa specimens showing MYBL2 expression relative to the level of RACGAP1 and nuclear YAP1 (χ^2^ test). ***P < 0.001. (C) Kaplan-Meier survival analysis of patients with PCa (n = 132). The log-rank test P-values are shown. (D) Model: overexpression of MYBL2 stimulates the YAP signaling through inducing RACGAP1-mediated RhoA activation, ultimately leading to castration-resistant growth and bone metastasis in PCa.

Finally, we evaluated the clinical relevance of the MYBL2 and YAP signaling pathways in human PCa specimens. IHC staining showed that MYBL2 expression was positively and significantly correlated with RACGAP1 expression (*P* < 0.001) and nuclear YAP expression (*P* < 0.001) (Figure 7A-B). Importantly, survival analysis revealed that patients with high expression of both MYBL2 and RACGAP1 or with high expression of MYBL2 and nuclear YAP had worse DFS than other groups among all patients with PCa (Figure 7C). Collectively, these results support the notion that overexpression of MYBL2 stimulates YAP signaling by inducing RACGAP1-mediated RhoA activation, ultimately leading to castration-resistant growth and poor prognosis in PCa (Figure 7D).

## Discussion

CRPC remains dependent on AR for growth and is characterized by aberrant reactivation of AR signaling 36, 37. However, further suppression of AR signaling using second-generation AR antagonists and inhibitors of androgen synthesis is not curative, and despite extending survival, disease progression is inevitable 5; this is because CRPC tumors are highly heterogeneous, with variable clinical outcomes, and CRPC progression might also involve bypass and compensatory signaling pathways 9, 38. In the present study, we identified MYBL2 as a potential driver of ADT resistance. Mechanistically, MYBL2 induced castration-resistant growth and bone metastasis of PCa cells by controlling Hippo-YAP signaling through RACGAP1-mediated RhoA activation. MYBL2 silencing or treatment with either the YAP/TAZ inhibitor Verteporfin or the RhoA inhibitor Simvastatin, reversed resistance to ADT and blocked bone metastasis of CRPC cells. Therefore, our results identified a new molecular mechanism conferring resistance to ADT and revealed potential therapeutic strategies against metastatic CRPC.

MYBL2 has been characterized as a putative oncogene in aggressive cancers 15, 16. MYBL2 promotes the malignant progression of tumors by controlling cancer cell proliferation, therapy resistance, and metastasis 19, 39, 40. Notably, MYBL2 is implicated in castration-resistant PCa growth 20, 41, 42. However, few previous studies have examined the specific functional and clinical implications of MYBL2 in CRPC. Herein, we found that MYBL2 expression was progressively upregulated in benign prostate tissues, primary or localized PCa, and metastatic CRPC, which is consistent with a previous report 18. Moreover, high MYBL2 expression was positively associated with TNM stage, total PSA levels, and Gleason score and predicted a higher risk of metastatic relapse and poor prognosis in patients with PCa, indicating that MYBL2 expression is a sign of aggressive PCa. Remarkably, MYBL2 overexpression in androgen-dependent PCa cells enhanced *in vitro* growth and anti-apoptosis in androgen-deprived conditions and facilitated castration-resistant growth and metastatic capacity* in vivo*. Thus, MYBL2 also functions as an oncogene in CRPC progression and exerts its effects by regulating the Hippo-YAP signaling pathway.

The regulation of MYBL2 itself is comprehensive and involves control of expression at the transcriptional, translational, and post-translational levels. Notably, amplification rates of MYBL2 transcription were found to be significantly increased during the development of hormone escape 43, suggesting that genomic amplification may be involved in MYBL2 dysregulation. Moreover, MYBL2 is also directly controlled at the transcriptional level by the dimerization partner, RB-like, E2F and multi-vulval class B (DREAM) multiprotein complex during the cell cycle 44. In addition, a previous study reported that miR-30a was downregulated in CRPC and could bind to the 3' UTR of MYBL2 mRNA to reduce its abundance 20. The protein level of MYBL2 is also modulated by post-translational modifications such as phosphorylation and acetylation 45, 46. Cyclin A/E-CDK2 phosphorylates MYBL2 and induces its transactivation activity, subsequently leading to ubiquitin-mediated proteolysis of MYBL2 protein 45. However, the molecular mechanism of MYBL2 dysregulation in CRPC requires further investigation.

In the present study, we found that MYBL2 could upregulate RACGAP1 expression by directly binding to the consensus MYBL2 binding sequence in the RACGAP1 promoter, consistent with previous reports, indicating that the transactivation of several pro-survival target gene promoters occurs via direct binding of MYBL2 to a consensus Myb-binding site (MBS) 11, 47-49. However, it was recently shown that the multi-vulval class B (MuvB) core dissociates from the DREAM complex upon cell cycle entry and sequentially recruits MYBL2 (MYBL2-MuvB complex) to bind to the promoters of G2/M genes during S phase and activates their transcription 44, 50. Notably, knockdown of either MYBL2 or components of the MuvB core prohibited the binding of both factors to the promoters of these genes 50. Consistently, mutation of either MBS or the cell cycle gene homology region (CHR, bound by LIN54 of the MuvB core) element in the promoters of these target genes independently disrupts the promoter activity of MYBL2 and the MuvB core 51, 52. These results show a dependency of both factors and their DNA-binding domains in transactivating late cell cycle genes. Thus, it is possible that RACGAP1 is regulated by the MYBL2-MuvB complex, and that MYBL2 is recruited to the RACGAP1 promoter, dependent on CHR promoter element-mediated binding of MuvB. Nevertheless, further research is necessary to validate this hypothesis.

Overexpression and/or nuclear translocation of YAP is relatively common in human cancers, suggesting constitutive activation of YAP/TAZ 53. The Hippo pathway is regulated by many biological mechanisms, including cell polarity, cell-cell contact, and mechanotransduction 54. Moreover, genetic and epigenetic modifications, or post-transcriptional regulation of Hippo signaling cascade members, as well as crosstalk with other signaling pathways such as G-protein-coupled receptor (GPCR) signaling contribute to Hippo pathway disturbance 55. Further understanding of the regulatory mechanisms of the Hippo-YAP pathway may identify novel therapeutic targets for PCa. In the present study, we found that MYBL2 transcriptionally activates RACGAP1, thereby disrupting the activity of LATS1 kinase and enhancing YAP-TEAD transcriptional programming by modulating RhoA. Interestingly, previous studies have revealed a connection between the YAP signaling pathway and the MMB complex subunit MYBL2 in the control of cell cycle progression 21, 22. Specifically, YAP induces MYBL2 gene expression and promotes the binding of MYBL2 to the promoters of several pro-proliferation genes to activate their transcription 21. The results of the present study extend our understanding of their connection by showing that MYBL2 also affects YAP-TEAD transcriptional activity by regulating the RACGAP1/RhoA/LATS1 kinase cascade, implying a positive feedback mechanism between MYBL2 and YAP signaling in tumor initiation and malignant progression.

The Hippo-YAP pathway is an important downstream branch of the mevalonate-RhoA GTPase signaling system 56. Simvastatin, a potent inhibitor of 3-hydroxy-3-methylglutaryl-CoA reductase, traditionally used in cardiovascular diseases to reduce lipid levels, could induce YAP phosphorylation by repressing Rho GTPase activity and actin rearrangement 57. Emerging evidence indicates that Simvastatin inhibits cancer cell proliferation, migration, and survival by regulating Rho GTPase activation 58. Verteporfin, traditionally used to treat age-related macular degeneration, is a small molecule inhibitor of YAP-TEAD interactions that potently suppresses YAP-induced oncogenic growth 59. Developing drugs that target oncogenic transcription factors or directly restore tumor suppressor kinase function has proven challenging. In the present study, we attempted to use the FDA-approved drugs Simvastatin and Verteporfin to achieve pharmacological inhibition of key nodes in the MYBL2/RACGAP1/RhoA/YAP signaling cascade and found that these treatment regimens were sufficient to reverse ADT resistance and the metastatic phenotype in CRPC cells. Consistent with our results, growing evidence supports the role of statins in reducing the risk of advanced and lethal PCa 60. Further clinical trials have shown that statins are promising candidates for adjuvant therapy with abiraterone in patients with metastatic CRPC and correlated with superior overall survival 61. In addition, both Simvastatin and Verteporfin are clinically applied drugs with few side effects, while Simvastatin is a low-cost drug. Our results provide evidence supporting the use of these drugs in PCa therapy. However, whether Simvastatin or Verteporfin treatment strategies are effective for patients with CRPC requires further investigation.

## Conclusions

Our study demonstrated that MYBL2 controls the development of castration resistance and metastatic relapse in PCa by modulating the Hippo-YAP signaling pathway by facilitating RACGAP1-mediated RhoA activation. We propose that therapeutic intervention centered on eliminating MYBL2 expression or inhibiting YAP/TAZ function as a consequence of dysregulated RhoA GTPase activity could be useful in preventing ADT resistance and blocking bone metastasis in PCa.

## Supplementary Material

Supplementary figures and tables.

## Abbreviations

ADT: Androgen-deprivation therapies
AR: Androgen-receptor
ATCC: American Type Culture Collection
ChIP: Chromatin immunoprecipitation
CRPC: Castration-resistant prostate cancer
DFS: Disease-free survival
GEO: Gene Expression Omnibus
GPCR: G-protein-coupled receptor
H&E: Hematoxylin and eosin
IHC: Immunohistochemistry
MYBL2: MYB proto-oncogene like 2
PRAD: Prostate Adenocarcinoma
qRT-PCR: Quantitative real-time PCR
RACGAP1: Rac GTPase activating protein 1
shRNA: Short hairpin RNA
TCGA: The Cancer Genome Atlas
TUNEL: Terminal deoxynucleotidyl transferase nick-end labeling
