# Supplementary material for: MYBL2 disrupts the Hippo-YAP pathway and confers castration resistance and metastatic potential in prostate cancer
Source: Theranostics. 2021 Mar 31;11(12):5794–812. doi: 10.7150/thno.56604 (PMC8058714; doi:10.7150/thno.56604)
Supplement: Supplementary file 1 — Supplementary figures and tables. [file thnov11p5794s1.pdf]

Supplementary Information

Supplementary Figures

Figure S1. Upregulation of MYBL2 correlates with advanced progression and poor prognosis in prostate cancer.

Figure S2. MYBL2 inhibits Hippo signaling leading to YAP activation.

Figure S3. MYBL2 significantly upregulates RACGAP1, a critical repressor of Hippo signaling.

Supplementary Tables

Table S1. Primers for real-time PCR analysis.

Table S2. Primers for ChIP-PCR assay.

Table S3. Correlation between MYBL2 expression and clinicopathological characteristics of patients with prostate cancer.

Table S4. Univariate and multivariate analysis of factors associated with disease-free survival in 132 patients with prostate cancer.

Supplementary Figures

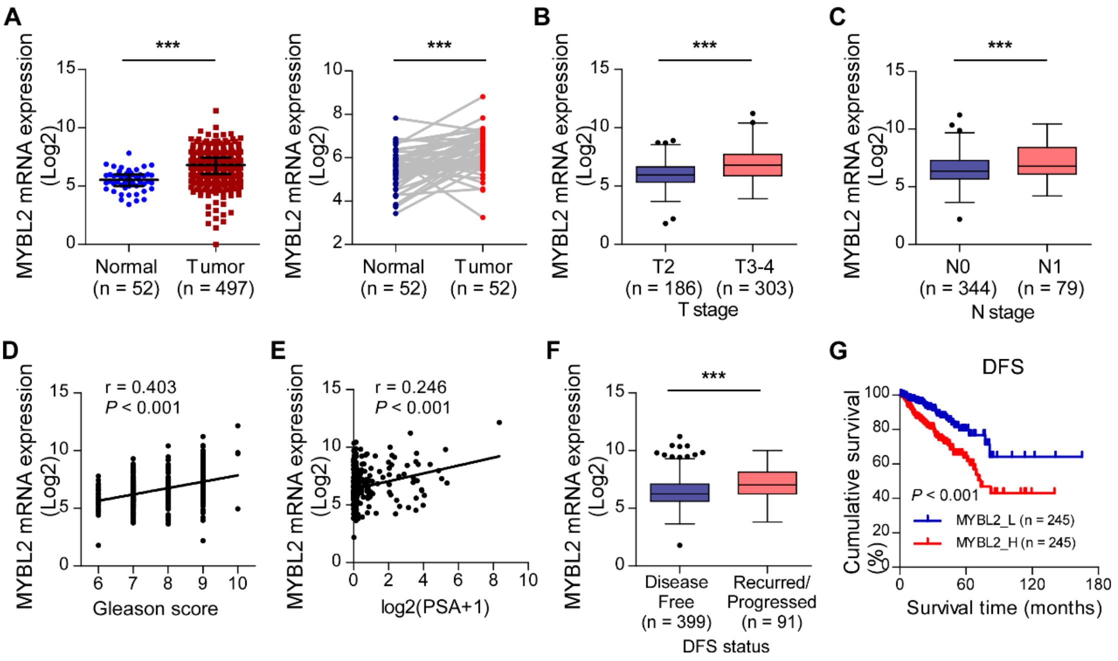

Figure S1. Upregulation of MYBL2 correlates with advanced progression and poor prognosis in prostate cancer. (A) MYBL2 mRNA expression in the prostate cancer tissues were assessed by analyzing the TCGA prostate adenocarcinoma dataset (PRAD, Normal, n = 52; Tumor, n = 497 (left panel, two-tailed Student's t test) and 52 paired normal tissues and prostate cancer tissues (right panel, paired Student's t test)). (B-C) MYBL2 mRNA expression in the prostate cancer tissues in TCGA (PRAD) dataset. (B) T stage: T2, n = 186; T3-4, n = 303. (C) N stage: N0, n = 344; N1, n = 79. (D-E) Scatter diagram and linear regression analysis of Pearson correlation between MYBL2 levels and Gleason score (D), and PSA (E) in TCGA (PRAD) dataset. (F) MYBL2 mRNA expression in the prostate cancer tissues with Disease Free (n = 399) or Recurred/Progressed (n = 91) in TCGA (PRAD) dataset. (G) Kaplan-Meier analysis of DFS curves for patients with prostate cancer with low MYBL2 expression (MYBL2_L; n = 245) versus high MYBL2 expression (MYBL2_H; n = 245) based on

an analysis of the TCGA (PRAD) dataset (log-rank test). In (B, C and F), *P*-values were determined by two-tailed Student's t test. In (D-E), r-values were determined by Pearson's test, *P*-values were determined by two-tailed Student's t test. ***$P < 0.001$.

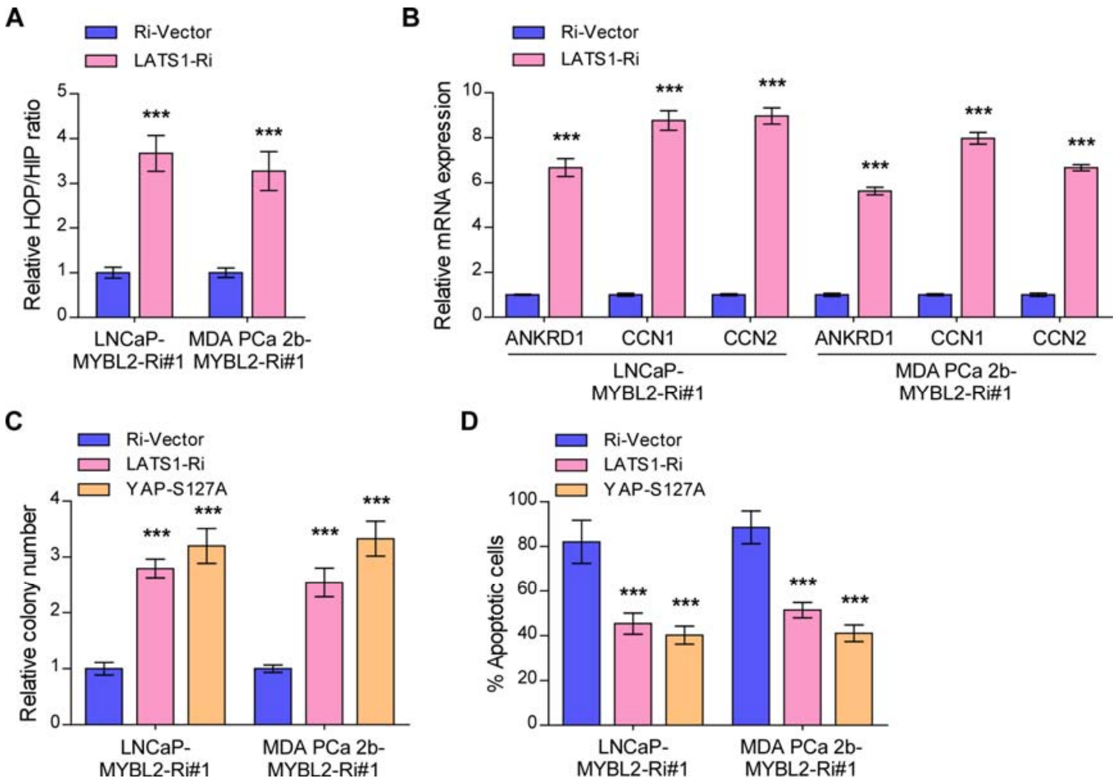

Figure S2. MYBL2 inhibits Hippo signaling leading to YAP activation. (A) Luciferase activity of the YAP/TAZ-TEAD promoter reporter was examined in the indicated cells. (B) QRT-PCR analysis of YAP1-targeted genes mRNA expression in the indicated cells. Gene expression levels were normalized to GAPDH. (C) Quantification of colony formed by the indicated cell lines cultured in medium with CSS. (D) Apoptosis assessed in the indicated cells treated in medium supplemented with 0.5% CSS for 4 days. *P*-values were determined by two-tailed Student's t test in (A and B) and One-way ANOVA test in (C and D). Data were presented as mean ± SD. ***$P < 0.001$.

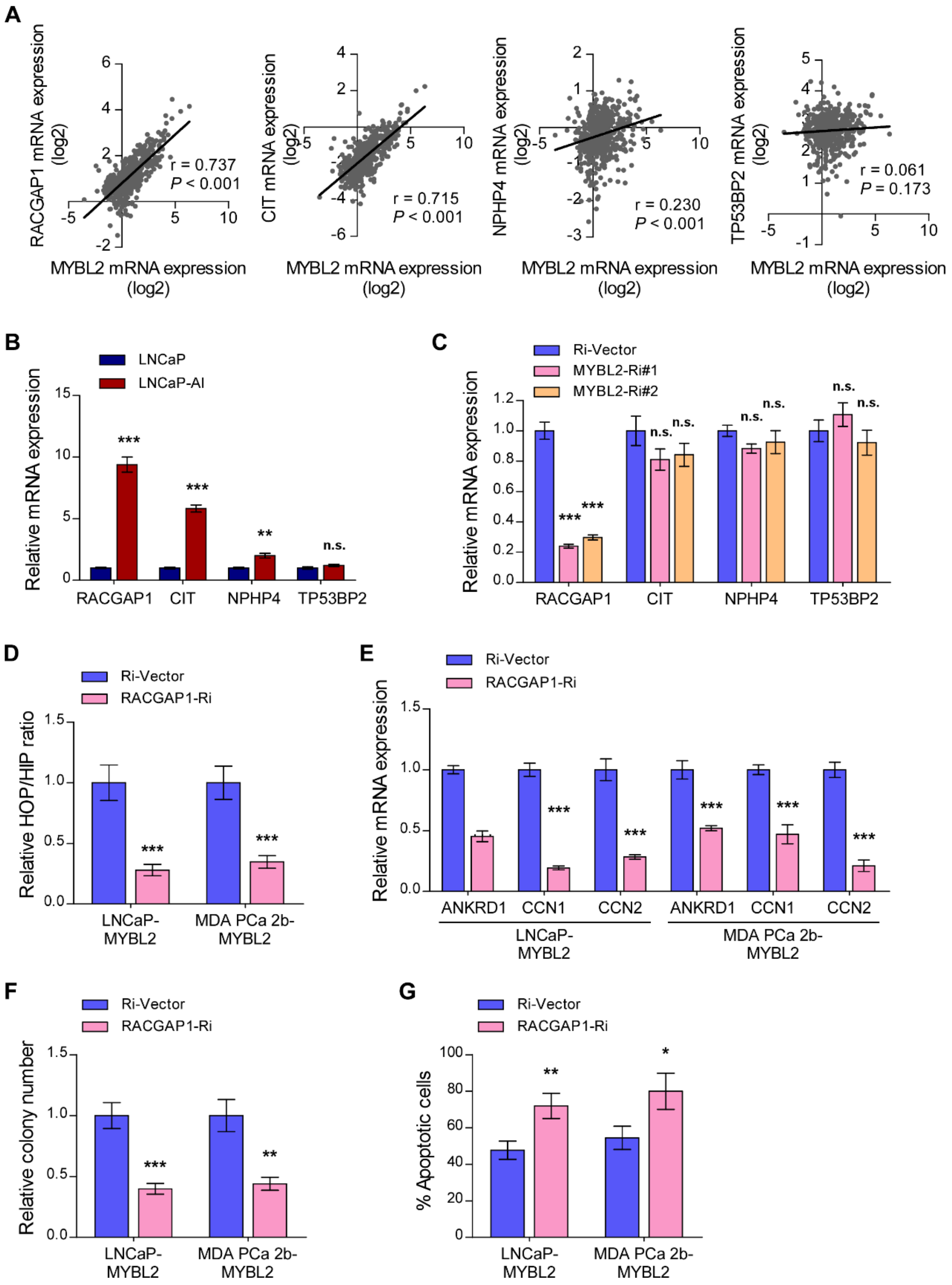

Figure S3. MYBL2 significantly upregulates RACGAP1, a critical repressor of Hippo signaling. (A) Scatter diagram and linear regression analysis of Pearson correlation between MYBL2 levels and the negative regulators of YAP1 (RACGAP1, CIT, NPHP4 and TP53BP2) in TCGA (PRAD) dataset. r-values were determined by

Pearson's test, P-values were determined by two-tailed Student's t test. (B) QRT-PCR analysis of RACGAP1, CIT, NPHP4 and TP53BP2 mRNA expression in the LNCaP and LNCaP-AI cells. Gene expression levels were normalized to GAPDH. Student's t test. (C) QRT-PCR analysis of RACGAP1, CIT, NPHP4 and TP53BP2 mRNA expression in the indicated groups of LNCaP-AI cells. Gene expression levels were normalized to GAPDH. One-way ANOVA test. (D) Luciferase activity of the YAP/TAZ-TEAD promoter reporter was examined in the indicated cells. (E) QRT-PCR analysis of YAP1-targeted genes mRNA expression in the indicated cells. Gene expression levels were normalized to GAPDH. (F) Quantification of colony formed by the indicated cell lines cultured in medium with CSS. (G) Apoptosis assessed in the indicated cells treated in medium supplemented with 0.5% CSS for 4 days. *P*-values were determined by two-tailed Student's t test in (D-G). Data were presented as mean ± SD. *$P < 0.05$, **$P < 0.01$, ***$P < 0.001$.

Supplementary Tables

Table S1. Primers for real-time PCR analysis.

Gene		Sequence (5'-3')
MYBL2	forward	CTTGAGCGAGTCCAAAGACTG
	reverse	AGTTGGTCAGAAGACTTCCCT
CCN1	forward	GGTCAAAGTTACCGGGCAGT
	reverse	GGAGGCATCGAATCCCAGC
HOXA1	forward	TCCTGGAATACCCCATACTTAGC
	reverse	GCACGACTGGAAAGTTGTAATCC
AMOTL2	forward	ACCATGCGGAACAAGATGGAC
	reverse	GGCGGCGATTTGCAGATTC
HOXC13	forward	CTCATCCCCGTCGAAGGCTA
	reverse	GCTGCACCTTAGTGTAGGGC
EDN1	forward	AGAGTGTGTCTACTTCTGCCA
	reverse	CTTCCAAGTCCATACGGAACAA
BIRC2	forward	GAATCTGGTTTCAGCTAGTCTGG
	reverse	GGTGGGAGATAATGAATGTGCAA
SOX6	forward	TACCTCTACCTCACCACATAAGC
	reverse	ACATCGGCAAGACTCCCTTTG
CCN2	forward	CAGCATGGACGTTCGTCTG
	reverse	AACCACGGTTTGGTCCTTGG
ANKRD1	forward	CGTGGAGGAAACCTGGATGTT
	reverse	GTGCTGAGCAACTTATCTCGG
AREG	forward	GAGCCGACTATGACTACTCAGA
	reverse	TCACTTTCCGTCTTGTTTTGGG
FGF2	forward	AGAAGAGCGACCCTCACATCA
	reverse	CGGTTAGCACACACTCCTTTG
BIRC5	forward	AGGACCACCGCATCTCTACAT

	reverse	AAGTCTGGCTCGTTCTCAGTG
RACGAP1	forward	TGCACGTAATCAGGTGGATGT
	reverse	TGAATCTGTCGTTCCAGCTTTT
GAPDH	forward	AAGGTGAAGGTCGGAGTCAA
	reverse	AATGAAGGGGTCATTGATGG

Table S2. Primers for ChIP-PCR assay.

Gene (promoter)		Sequence(5'-3')
RACGAP1-P1	forward	CTACGGTGTGACGTACGCG
	reverse	CAGGAAGTACCACTTGCCCTTAG
RACGAP1-P2	forward	TCAGGATGCCATATTGACTAAGG
	reverse	ATGGAAGGGATAATATAGCTCAGTG

Table S3. Correlation between MYBL2 expression and clinicopathological characteristics of patients with prostate cancer.

Characteristics		Total	MYBL2		*P* value
			Low expression	High expression	
Age (years)	≤ 73	63	37	26	0.356
	> 73	69	35	34	
T stage	T2	74	51	23	< 0.001
	T3–4	58	21	37	
N stage	N0	84	55	29	< 0.001
	N1	48	17	31	
Total PSA (ng/ml)	≤ 10	60	39	21	0.028
	> 10	72	33	39	
Gleason score	≤ 7	61	39	22	0.045
	> 7	71	33	38	
Relapse/Metastasis status	Negative	98	62	36	< 0.001
	Positive	34	10	24	

Table S4. Univariate and multivariate analysis of factors associated with disease-free survival in 132 patients with prostate cancer.

Characteristics	Univariate analysis		Multivariate analysis	
	HR (95% CI)	*P* values	HR (95% CI)	*P* values
Age (years, > 73)	1.069 (0.542-2.111)	0.847	1.310 (0.614-2.793)	0.485
T stage (T3–4)	1.278 (0.651-2.508)	0.477	1.891 (0.888-4.028)	0.099
N stage (N1)	3.026 (1.497-6.115)	0.002	2.372 (1.050-5.356)	0.038
Total PSA (ng/ml, > 10)	2.216 (1.057-4.646)	0.035	1.965 (0.928-4.160)	0.078
Gleason score (> 7)	2.344 (1.128-4.868)	0.022	1.741 (0.810-3.740)	0.156
MYBL2 expression (high)	4.298 (2.040-9.055)	<0.001	3.427 (1.505-7.806)	0.003

HR, hazard ratio; CI, confidence interval.
